# A landscape of metallophore synthesis and uptake potential of the genus *Staphylococcus*

**DOI:** 10.1093/nargab/lqaf183

**Published:** 2025-12-17

**Authors:** Mathias Witte Paz, Alina Bitzer, Kay Nieselt, Simon Heilbronner

**Affiliations:** Institute for Bioinformatics and Medical Informatics, University of Tübingen, Tübingen, 72076, Germany; Interfaculty Institute of Microbiology and Infection Medicine, University of Tübingen, Tübingen, 72076, Germany; Biozentrum, Ludwig-Maximilians-University München, Munich, 80539, Germany; Institute for Bioinformatics and Medical Informatics, University of Tübingen, Tübingen, 72076, Germany; Interfaculty Institute of Microbiology and Infection Medicine, University of Tübingen, Tübingen, 72076, Germany; Biozentrum, Ludwig-Maximilians-University München, Munich, 80539, Germany

## Abstract

Metallophores are secondary metabolites that enable bacterial growth in metal-limited environments such as the human nasal microbiome. While synthesis and uptake of metallophores in *Staphylococcus aureus* are well characterized, the diversity across the *Staphylococcus* genus remains unclear. We performed a comprehensive bioinformatic analysis of 77 representative species, as well as over 1800 strains, to map metallophore biosynthetic gene clusters (BGCs) and uptake systems. Staphyloferrin A (SF-A) biosynthesis was widely conserved, though disrupted loci were found in some species, with some of them appearing to have replaced SF-A with a newly discovered, still uncharacterized, BGC. In contrast, staphyloferrin B and staphylopine production were restricted to select species. Uptake systems were more broadly distributed, showing evidence of “cheating” species that lack biosynthesis, but retain the required lipoproteins for metallophore usage. *Staphylococcus lugdunensis* exemplifies this, encoding multiple uptake systems without producing known metallophores. Strain-level variation was also observed, particularly with specific cases of SF-A truncation, but also for the diversity of lipoprotein receptors. These findings highlight the diversity of metallophore systems, suggesting diverse metallophore-dependent cooperation and competition within the *Staphylococcus* genus. This work provides a foundation for future experimental studies to identify the role of metallophores in microbial community interactions.

## Introduction

*Staphylococci* are common members of the human and animal microbiota. The pathogen *Staphylococcus aureus* colonizes the anterior nares of approximately one-third of the human population, and this colonization represents a major risk factor for subsequent infections [1, 2]. Other staphylococcal species generally considered less virulent—such as *Staphylococcus epidermidis*—are ubiquitously present on human skin and nasal mucosa [3].

Skin and mucosal surfaces are nutrient-limited environments, particularly with respect to trace metal ions such as iron (Fe), manganese (Mn), copper (Co), and zinc (Zn). These transition metals are essential cofactors for key metabolic enzymes, and although required only in trace amounts, bacterial growth is fully dependent on their availability. Among them, iron has been studied most extensively due to its critical role in enzymes involved in DNA replication, glycolysis, and respiration [4, 5]. However, Mn and Zn are also essential for bacterial physiology [6]. On epithelial surfaces, host-secreted metal-binding proteins such as lactoferrin (Fe) and calprotectin (Mn/Zn) restrict the availability of these ions [7]. This defense mechanism is known as nutritional immunity and limits bacterial proliferation [8].

To overcome metal starvation, many microorganisms possess dedicated biosynthetic gene clusters (BGCs) allowing the production of metallophores, small, high-affinity secondary metabolites that scavenge trace metals from the environment. In some cases, metallophores even extract metals directly from host proteins like lactoferrin or calprotectin [9]. Among the best-studied classes of metallophores are siderophores, a subclass specialized in iron scavenging [10]. Once metal-saturated, the chelators are recognized by specific receptors on the bacterial surface (lipoproteins in Gram-positive bacteria) and imported into the cell, where the metal is released via reduction or degradation of the metal chelator. Metallophores can function as public goods within bacterial communities. Once secreted, they can be utilized by neighboring bacteria that express compatible receptors. This enables diverse ecological interactions, ranging from cooperative “shared labor” to exploitative “cheating,” where non-metallophore-producing bacteria steal the benefits without contributing to the metabolic costs of their production [11].

**Figure 1.**
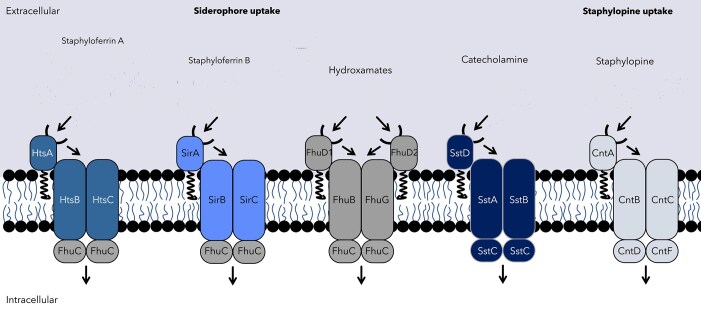
Schematic representation of *S. aureus* siderophores and metallophores, as well as their acquisition systems.

*Staphylococcus aureus* is known to produce metallophores, and both its metallophores and their physiological relevance are well characterized [12] (Fig. 1). The pathogen synthesizes the iron-binding siderophores staphyloferrin A (SF-A) and staphyloferrin B (SF-B). SF-A is a carboxylate-type siderophore produced via a nonribosomal peptide synthesis (NRPS)-independent system: The BGC consists of *sfaABCD* genes, with *sfaB/D* representing *iucA/C* homologs, making SF-A a classical NRPS-independent siderophore (NIS) [13]. Iron-loaded SF-A is recognized by the substrate-binding lipoprotein HtsA and imported via the HtsABC system [14]. SF-B is also a carboxylate-type siderophore, synthesized by SbnABCDEFGHI in an NRPS-independent fashion but without IucA/C homologs [15]. The siderophore is then imported via the SirABC system, following recognition by the SirA lipoprotein [16].

In addition to iron acquisition, *S. aureus* produces the Mn/Zn-binding metallophore staphylopine (STP), which is also synthesized in an NRPS-independent manner by CntKLME [17]. Metal-saturated STP is recognized by the lipoprotein CntA and imported through the CntABCDF system.

*Staphylococcus aureus* is also capable of utilizing siderophores produced by other organisms (xenosiderophores). The lipoproteins FhuD1 and FhuD2 mediate uptake of hydroxamate-type siderophores [18, 19]. In contrast to HtsA, SirA , and CntA, the xenosiderophore receptors are more promiscuous and more than one dedicated molecule can be recognized. The precise structural mechanism of hydroxamate binding to the FhuD1/FhuD2 receptors is unclear, but it has been shown that the proteins bind different hydroxamate-type siderophores with different affinities, suggesting specialization and multiple alleles might allow an increased spectrum of hydroxamates to be acquired [19, 20]. Similarly, SstA can recognize catecholate-type iron chelators from host and bacterial origin [21], and, as identified in *S. lugdunensis*, different alleles are proposed to expand the range of molecules that can be acquired [22].

While SF-A is thought to be widely produced across the staphylococcal genus, SF-B production appears to be restricted to *S. aureus* [15]. However, the capacity of other staphylococci to produce metallophores or to use those of other species remains poorly understood. Additional insight is needed to enhance our understanding of metallophore-driven interspecies interactions within the human microbiome.

In this study, we conducted a comprehensive bioinformatic analysis of metallophore biosynthesis and acquisition genes across the *Staphylococcus* genus. We found that while SF-A biosynthetic genes are broadly conserved, several species possess truncated loci, likely rendering them non-functional. Interestingly, one distinct phylogenetic clade harbors a metallophore biosynthesis operon that remains to be characterized, possibly compensating for the loss of SF-A production. SF-B biosynthetic genes were confined to *S. aureus* and closely related, animal-associated species. However, several coagulase-negative staphylococci (CoNS) encode the SF-B acquisition gene *sirA*, suggesting an adaptation to exploit SF-B produced by pathogens. Likewise, xenosiderophore uptake genes (*fhuD, sstA*) are widespread, and many species carry multiple paralogs, indicating functional diversification and expanded substrate specificity. Finally, we observed low intra-species variation in metallophore biosynthesis and acquisition genes, suggesting that horizontal gene transfer of these systems is limited within this genus.

## Materials and methods

To characterize the metallophore synthesis and uptake mechanisms in the staphylococcal genes, we conducted an analysis on the species and on the strain level. For the analysis on the *Staphylococcus* species level, the accession numbers of representative samples for each *Staphylococcus* species were collected from the Genome Taxonomy Database (GTDB) [23]. This resulted in 84 genomes that span the available species. For some species, the GTDB returned more than one representative. In these cases, only one representative per species was selected for a more consistent comparison, resulting in 77 genomes (Supplementary Table S1).

For the analysis on the strain level, four human-related species were selected. The accession numbers of all available strains from the GTDB were retrieved, and the corresponding genomes and protein sequences were downloaded from the NCBI database. Some strains’ assemblies were removed due to their low quality (N50 < 50 kb or they contained a total of >250 contigs). In total, ~1800 strains were analyzed. The final number of strains per species for this analysis can be found in Table 1. The corresponding accession codes can be found as supplementary data.

**Table 1.** Number of available strains from the GTDB and number of remaining assemblies after filtering for four selected species and quality (N50 $> 50$ kb, #contigs < 250)

Species	Number of strains retrieved from the GTDB	Used assemblies
*S. capitis*	215	180
*S. epidermidis*	1556	1292
*S. hominis*	274	235
*S. lugdunensis*	144	128

The following analyses were first computed for the set of species and applied subsequently on the selected strains. Based on the retrieved genomes, BGCs were identified using antiSMASH (version 7.1.0) [24]. antiSMASH classifies the BGCs into different cluster types with a rough functional description. Due to our focus on siderophore synthesis potential, we focused only on BGCs labeled as NRPS-independent siderophores and nonribosomal peptide (NRP) metallophores. Additional modules from antiSMASH were activated to annotate the identified BGCs. The module ClusterCompare annotates and provides a similarity score to BGCs found in the MIBiG database [25]. If a BGC was similar to any known metallophore-related BGC in *S. aureus* (*sfaABCD, sbnABCDEFGHI*, or *cntKLME*), we quantified its completeness. That is, we assessed whether all required synthesis genes were present. The BGCs were classified as complete if they had $100\%$ similarity to the annotated counterpart. Otherwise, they were considered incomplete as long as the similarity was not zero. If the analyzed representative for a species showed an incomplete BGC, other available strains from the GTDB were analyzed to assess whether this was a species-wide observation.

Lastly, while some BGCs were classified as metallophores or siderophores, they reflected no similarity to any BGC in the MIBiG database via ClusterCompare. These BGCs were then compared to each other and to the reference BGCs of *S. aureus* using the tool clinker (version 0.0.31) [26].

Since antiSMASH focuses solely on the identification of biosynthetic genes, it cannot assess a species’ capacity to uptake metallophores. In *Staphylococci*, lipoproteins play a key role in the uptake of siderophore-iron complexes. Hence, we expect that gene homologs are present in the investigated genomes and can uptake the corresponding siderophore. Considering the evolutionary diversity across species and strains, our objective was to establish a threshold that would yield significant outcomes in terms of sequence diversity for homolog identification.

To analyze the uptake potential of the species, a sequence homology analysis with MMSEQS2 (version 15, commit: ad6dfc) was performed [27]. The amino acid sequence of the six lipoproteins of *S. aureus* was used as queries in MMSEQS2 to search within all staphylococcal proteomes. To identify the best threshold, the results for the *S. aureus* search were analyzed in detail. After removing the self-hits ($100\%$ similarity), the highest off-target similarity was observed between HtsA and a heme-binding protein, at $49.2\%$ similarity. Based on this, we set a $50\%$ sequence similarity threshold to define potential homologs of the protein references across the remaining species. If a protein shows a similarity higher than $50\%$ to more than one of the reference lipoproteins of *S. aureus*, the lipoprotein was called homolog only to the reference gene with the highest similarity. Moreover, multiple genes of the same species can be identified as potential homologs of one reference lipoprotein. In such cases, the number of potential homologs was counted and the similarity value of the best hit per lipoprotein per species is retained.

To identify whether specific phylogenetic clusters had similar metallophore synthesis and uptake potential, a phylogenetic tree was calculated based on a core-genome multilocus sequence typing (cgMLST). The cgMLST was identified using chewBBACA (version 3.3.10) [28] to then create a distance matrix using the script mlst2dist (https://github.com/tripitakit/mlst2dist) based on the algorithm proposed by Allelematch [29]. From the resulting distance matrix, a phylogenetic tree was reconstructed using the neighbor-joining algorithm implemented in MEGA11 (version 11.0.11) [30].

For the synthesis potential at the strain level, we applied the same classification as before, categorizing strains as complete or incomplete based on the presence of the full BGC. In the case of *S. lugdunensis*, a previous study identified an NRPS system with metallophore potential [31]. To analyze the occurrence of this BGC across *S. lugdunensis* strains, we queried the BGCs identified by antiSMASH using BiG-SCAPE (version 2) [32] and included its presence in the summarized results. For the uptake potential, a strain was considered positive for uptake if it harbored at least one potential homolog of the reference lipoproteins, using the previously defined sequence similarity threshold of $50\%$. The results for metallophore synthesis and uptake potential were summarized using an UpSet plot [33]. The UpSet plot visualizes the distribution and overlap of these traits across strains for each species individually.

## Results and discussion

### Metallophore biosynthesis genes vary between staphylococcal species

**Figure 2.**
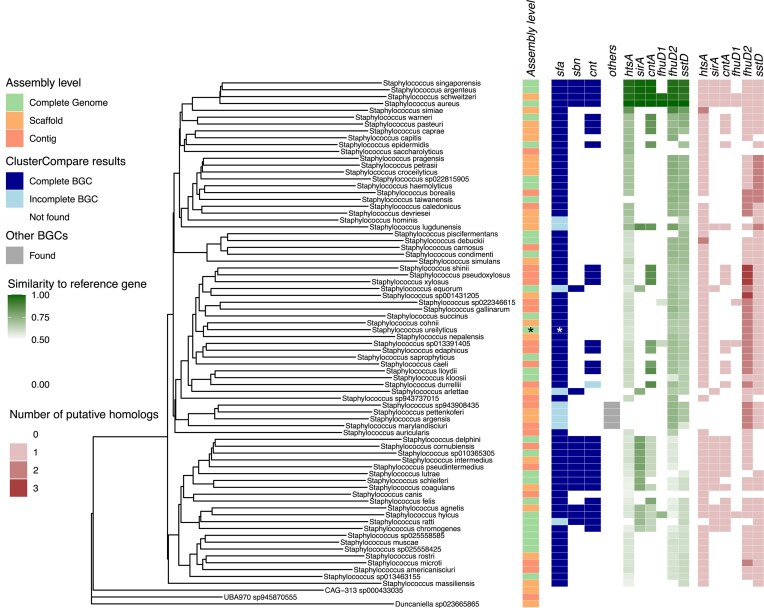
Overview of metallophore synthesis and uptake potential in the genus *Staphylococcus*. The dendrogram shows the phylogenetic relationships of staphylococcal species, alongside the results summarizing their metallophore-related biosynthetic and uptake capacities. The assembly level of each used reference is visualized in the first heatmap. The synthesis potential was assessed with antiSMASH. Results annotated as the three known *S. aureus* metallophores obtained with ClusterCompare are shown in the second-to-left heatmap column. Blue rectangles indicate BGCs, with dark blue representing complete BGCs (all synthesis genes present) and light blue indicating incomplete BGCs. Gray rectangles mark additional BGCs predicted as potential metallophore synthesizers but without specific annotation. The last two columns represent the uptake potential: green shading indicates the sequence similarity of the proteome to *S. aureus* metallophore uptake lipoproteins, while red shading shows the number of putative homologs identified. Strains that were reassessed and that have shown different results have been marked with an asterisk (*).

To investigate the genus *Staphylococcus*, we collected a non-redundant set consisting of 77 species representing all currently available species at the GTDB. A phylogenetic tree was computed based on the core genome MLST, and the presence of the *sfa, sbn*, and *cnt* gene clusters—associated with the production of SF-A, SF-B, and STP, respectively—was assessed (Fig. 2).

Though described as representatives for the corresponding species, the assemblies show a high diversity in their assembly levels (Fig. 2, leftmost heatmap column). Moreover, despite being classified as *Staphylococcus* in the NCBI taxonomy, our phylogenetic analysis identified three species as outliers, consistent with alternative genus-level classification proposed by the GTDB. Still, these species were included in our analysis and used as outgroups for the phylogenetic tree. It is important to note that none of these three species was identified as a potential metallophore producer or cheater by our analysis.

**Figure 3.**
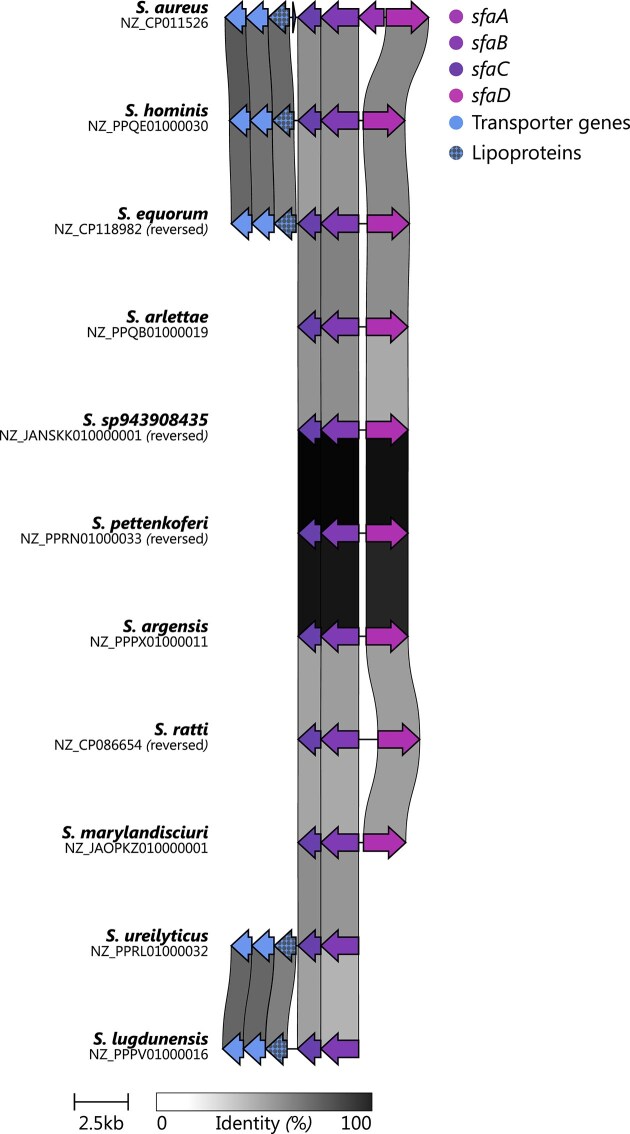
Comparison of all incomplete BGCs related to the synthesis of SF-A and the reference BGC of *S. aureus* using the tool clinker. The core biosynthetic genes (*sfaABCD*) are colored individually. Transporter genes are colored with respect to the annotation provided by antiSMASH. Other genes have been removed from the figure for clarity.

Our data show that SF-A biosynthesis is a core trait across the genus, with *sfa* genes present in all analyzed staphylococcal species (Fig. 2, leftmost blue heatmap column). However, 10 species carry truncated *sfa* loci, suggesting abrogation of SF-A biosynthesis. We used clinker to investigate structurally distinct *sfa* loci in more detail (Fig. 3). This analysis confirmed that *S. lugdunensis* harbors a truncated locus lacking *sfaDA* genes. This has been described previously and *in vitro* evidence is available showing that the truncation hinders SF-A biosynthesis [34]. Interestingly, *S. ureilyticus* (GCF_002902235.1) appears to carry a similar truncation although the location of the sequence at the very end of a contig raises some doubt regarding the accuracy or quality of this assembly. To address this, we repeated the analysis using the reference assembly from the RefSeq database (GCF_025558845.1) [35], which revealed a complete siderophore biosynthesis cluster. Nevertheless, since siderophore production is known to be strain-specific [36], the current assembly data do not allow for generalization of these findings to the entire species.

Additional eight species, including a clade containing *S. pettenkoferi, S. argensis, S. marylandensis*, and *S. sp943908435*, as well as individual species in distinct clades such as *S. equorum* and *S. hominis*, showed internal deletion of *sfaA* (Fig. 3). Analysis of additional genomes from *S. pettenkoferi, S. argensis, S. equorum*, and *S. hominis* with antiSMASH confirmed that *sfa* inactivation is conserved at the species level (Fig. 6B and Supplementary Table S2). Our results are in general agreement with a previous study, showing *S. chromogenes* but not *S. equorum* to produce SF-A [37].

Together, these data show that *sfa* genes are omnipresent within staphylococci, but recombinations of the locus are observed in 10 out of 74 ($13.5\%$) species, most likely hindering the production of SF-A.

In contrast, only $23\%$ of the species (17 genomes out of 74) harbor the *sbn* genes for SF-B biosynthesis. These include *S. aureus* and its close relatives (*S. schweitzeri, S. argenteus*, and *S. singaporensis*), but also more distantly related, non-human-associated species such as *S. equorum, S. delphini, S. pseudintermedius*, and *S. ratti*. This is in agreement with studies suggesting occasional SF-B production by non-*S. aureus* staphylococci [38].

**Figure 4.**
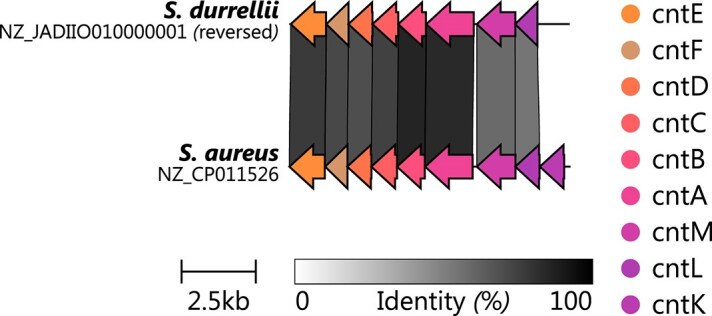
Comparison of incomplete *cnt* BGCs of *S. durrellii* to the corresponding loci of *S. aureus* using clinker. Other genes found within the BGC have been removed for clarity.

STP biosynthesis genes (*cnt*) are found in $39\%$ of species (29 out of 74) across various clades. One potentially nonfunctional *cnt* locus was identified in *S. durrellii*. Since no further genomes for this species are currently available at GTDB nor RefSeq, this hinders a further confirmation analysis for this species. However, we performed a detailed comparison of the *cnt* BGCs of *S. aureus* and *S. durrellii* (Fig. 4). This analysis revealed that *S. durrellii* lacks the gene *cntK*, most likely preventing STP production.

**Figure 5.**
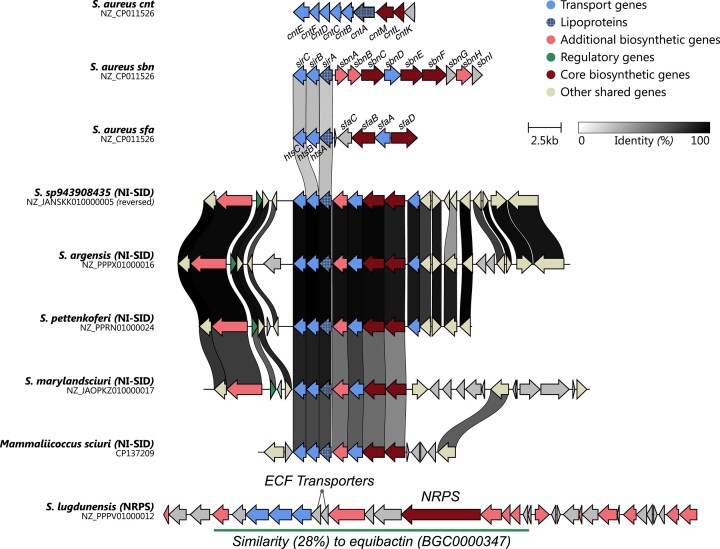
Comparison of novel BGCs identified in this study, known metallophore BGCs of *S. aureus*, and two uncharacterized BGCs from other studies using the tool clinker. For all BGCs, the genes have been colored with respect to the annotations provided by antiSMASH. For the last BGC, a subset of genes shows a similarity to the BGC producing equibactin (BGC0000347).

antiSMASH identified BGCs potentially involved in metallophore production that have not yet been annotated in the MIBiG database (Fig. 2, gray column). These novel NIS-BGCs were discovered in a clade comprising *S. pettenkoferi, S. argensis, S. marylandensis*, and *S. sp943908435*. For further characterization, the BGCs were compared to each other and to known metallophore-producing BGCs of *S. aureus* using clinker (Fig. 5). Moreover, two other BGCs previously associated with metallophore production were included in the comparison: an NRPS system in *S. lugdunensis* [31] and an NRPS-independent siderophore of *Mammaliococcus sciuri* (GCA_037849205.1, previously named *Staphylococcus sciuri*) [36].

The analysis showed a high degree of conservation between the uncharacterized clusters regarding both operon organization and sequence similarity, strongly suggesting that the strains produce the same or at least very similar compounds. Among these, the *S. marylandsciuri* BGC showed the highest sequence divergence, despite preserving the overall operon structure. Interestingly, the BGC identified in *M. sciuri* shows conserved synthesis and transporter genes, suggesting a resemblance to the uncharacterized BGCs of the mentioned clade. Importantly, no significant similarity to *sfa, sbn*, or *cnt* gene clusters was identified, suggesting that these BGCs likely encode the biosynthesis of a distinct, still uncharacterized metallophore. Notably, the phylogenetic clade of staphylococci carrying these BGCs corresponds to the *sfa*-deficient clade described above (Figs 2 and 3). Accordingly, it is tempting to speculate that this clade replaced SF-A with a novel siderophore, most likely to produce a privatized compound that is not shared with other staphylococcal species [11].

Furthermore, our analysis of *S. lugdunensis* also identified the previously reported NRPS system of unknown function [31]. This system shows no similarity to any other staphylococcal metallophore BGCs (Fig. 5), but antiSMASH shows a sequence similarity to the streptococcal NRPS system responsible for the production of the siderophore equibactin [39]. Nevertheless, it is important to highlight that the BGC is not annotated as a metallophore BGC, but only as an NRPS system; hence, it was not identified by our first analysis using antiSMASH. As discussed in the original publication, it is unclear if this system produces an antibacterial compound similar to lugdunin [40] or if it produces a metallophore. However, the similarity to equibactin along with the presence of a dedicated import system of the energy coupling factor type (ECF-transporter)—which is known as trace nutrient acquisition system [41]—strongly suggests the production of a nutrient scavenging molecule. Nevertheless, the observation that *S. lugdunensis* does not produce iron-scavenging siderophores *in vitro* [34, 36] might either suggest inactivity of the system or that the molecule does not target iron but a different metal such as Zn, Mn, or Cu. Additional laboratory-based experiments are needed to answer these questions.

### Metallophore acquisition systems vary between staphylococcal species

Metallophores, once secreted, act as public goods and can be utilized by other community members. To assess the potential for metallophore acquisition across the genus, we analyzed the distribution of genes encoding known siderophore receptors (Fig. 2, green columns).

The *htsA* gene, encoding the SF-A receptor, is broadly conserved, consistent with the widespread presence of *sfa* biosynthetic genes. In species lacking intact *sfa* loci, *htsA* was often also absent, supporting the idea of coordinated loss of synthesis and uptake systems. However, some species retained *htsA* despite nonfunctional *sfa* loci, suggesting that they may scavenge SF-A produced by other species. For example, SF-A usage of *S. lugdunensis* has been demonstrated experimentally [34]. Furthermore, it has been shown that SF-A is a widely accessible siderophore within the nasal microbiome and its usage by “cheaters” that do not produce siderophores themselves reduces the fitness of the producers [36]. Intriguingly, *S. taiwanensis* was the only species with an intact *sfa* operon but no detectable *htsA*. Since no additional genome sequences are currently available, the results derived with MMSEQS2 were analyzed in more detail. The most similar gene of *S. taiwanensis* (locus tag: HYI43_00815) to *htsA* has a similarity of around $40\%$, hence falling below our defined homology threshold of $50\%$. Interestingly, this gene is not found within the borders of the *sfa*-related BGC identified by antiSMASH, but lies ~680 kb from the core biosynthesis genes. Moreover, we evaluated the presence of the remaining components of the *htsABC* and identified homolog genes to *htsB* (HYI43_04060, $72.5\%$) and *htsC* (HYI43_04065, $73.8\%$). This led to the identification of a neighboring coding sequence region (HYI43_04055) with a substrate-binding protein potential found. However, this coding region is labeled as a pseudogene, explaining why the corresponding amino acid sequence was missing from the species’ proteome and hence from the MMSEQS2 analysis. Moreover, the sequence of this pseudogene translates to a 34 aa long protein sequence, highlighting the difference from the complete sequence of *htsA* of 327 aa. This raises questions about the functionality of this putative protein and therefore about *S. taiwanensis*’ ability to re-import SF-A.

All SF-B-producing species encode the corresponding receptor SirA (Fig. 2, second-left green column). Only two species, *S. lugdunensis* and *S. chromogenes*, were identified that encode *sirA* without an *sbn* cluster. Again, it has been experimentally demonstrated that *S. lugdunensis* can utilize SF-B and a similar trait can be expected for *S. chromogenes* [34]. Interestingly, the presence of *sirA* in *S. chromogenes* was shown to be strain-rather than species-specific [37] and the phenomenon of SF-B cheating appears to be a rare trait among staphylococcal species.

A similar picture was apparent for STP production and acquisition (Fig. 2, middle green column). Almost all species with STP biosynthesis genes harbor the corresponding receptor *cntA*, or, if they have an inactive locus, they retain the receptor. Interesting exceptions are *S. schleiferi*, which shows an intact *cntKLME* operon but no *cntA* receptor, and *S. lugdunensis*, which encodes *cntA* without corresponding biosynthesis genes. To verify the results of *S. schleiferi*, we proceeded similarly as with *S. taiwanensis*. Here, no other protein was identified as similar to *cntA* by our MMSEQS2 search. For the ABC transporters *cntBC*, two potential homologs were identified: JM183_RS11640 for *cntB* (sequence similarity: $64.4\%$) and JM183_RS11635 for *cntC* ($69.1\%$). A neighboring gene (JM183_RS11645) described as *nickel ABC transporter substrate-binding protein* was also identified in this case. However, a premature stop codon leads to a protein sequence of 390 aa, hence differing with respect to the expected length of 532 aa for *cntA*, suggesting that *S. schleiferi* might not be able to uptake STP.

Siderophores are costly metabolic molecules and act as public goods [11]. *Cheaters* arise within bacterial communities that consume but do not produce siderophores, which exhibit a metabolic burden to producers. Accordingly, siderophore production genes along with associated receptors are known to be under evolutionary pressure. For *Pseudomonas aeruginosa* it is known that the siderophore pyoverdine is produced with many subtle modifications, requiring modified receptors for acquisition [42]. This allows production of privatized siderophores preventing their usage by cheaters. It is widely unclear if this phenomenon is also occurring in *Staphylococci*.

To address this, we investigated the conservation of the metallophore-binding sites within the receptors HtsA, SirA, and CntA across species. A multiple sequence alignment was computed using ClustalO (v1.2.4) [43] for the sets of homologs across all species and the conservation of amino acid residues known to be engaged in binding of the metallophores [44–46] was analyzed (Supplementary Table S3). For HstA we found six out of nine important amino acids to be strictly conserved amongst the HstA alleles of all species. However, 21 species (33%) showed conserved divergence of three amino acids (R86N, K203Q, R304K), and a single species showed a unique profile (R86N, K203E, R304K) (Supplementary Fig. S1). It is tempting to speculate that these receptor variants might possess altered affinity toward SF-A or even recognize a modified version of the siderophore as observed for *P. aeruginosa* [42]. However, additional biological and biochemical evidence is needed to support this idea. For SirA, a divergence was observed in two species at two binding sites (Supplementary Fig. S2). An exchange of T144N was found in *S. lugdunensis*, and R206C was identified in *S. chromogenes* (Supplementary Fig. S2). The first species *S. lugdunensis* was demonstrated to consume SF-B, strongly suggesting that the receptor variant is able to recognize SF-B; putative effects on affinity remain, however, speculative. The effect of R206C on *S. chromogenes* remains unclear. Lastly, 55% of the identified CntA homologs differ from the canonical *S. aureus* receptor by carrying an N448T exchange (Supplementary Fig. S3). Again, it remains unclear if these exchanges affect specificity or affinity.

*Staphylococcus aureus* is known to import hydroxamate-type siderophores via the Fhu system [47], using two receptors, FhuD1 and FhuD2 [20]. Homologs of *fhuD1* were rare (5 out of 77 species), while *fhuD2* was widely conserved across the genus ($89\%$ of species, 69 out of 77, Fig. 2, second-right green column). Interestingly, several species harbored up to three distinct *fhuD2* homologs (Fig. 2, red columns), possibly expanding their hydroxamate siderophore uptake range.

The Sst system enables *S. aureus* to import catecholate-type siderophores [21]. Homologs of *sst* were widespread (67 out of 77, $87\%$, Fig. 2, rightmost green column). However, a clade of seven species, closely related to *S. lugdunensis*, harbored multiple *sst* copies. This duplication, previously observed in *S. lugdunensis* [22], may broaden the catecholate substrate range available to these species.

Taken together, *S. lugdunensis* presents a staphylococcal species with special characteristics. It appears to produce a single metallophore of so far unknown specificity. It produces neither SF-A nor SF-B nor STP but encodes the dedicated uptake systems for all these metallophores. Finally, the species encodes FhuD and multiple homologues of SstD. Altogether this suggests that *S. lugdunensis* is a highly adapted “cheater” that depends strongly on the biosynthetic potential of other species to thrive under metal-limited conditions.

### Strain-level variation

Recent studies have demonstrated that metallophore production and uptake may vary at the strain level [36]. To investigate this, we analyzed metallophore-related loci at the strain level for four selected species (*S. hominis, S. epidermidis, S. capitis*, and *S. lugdunensis*).

**Figure 6.**
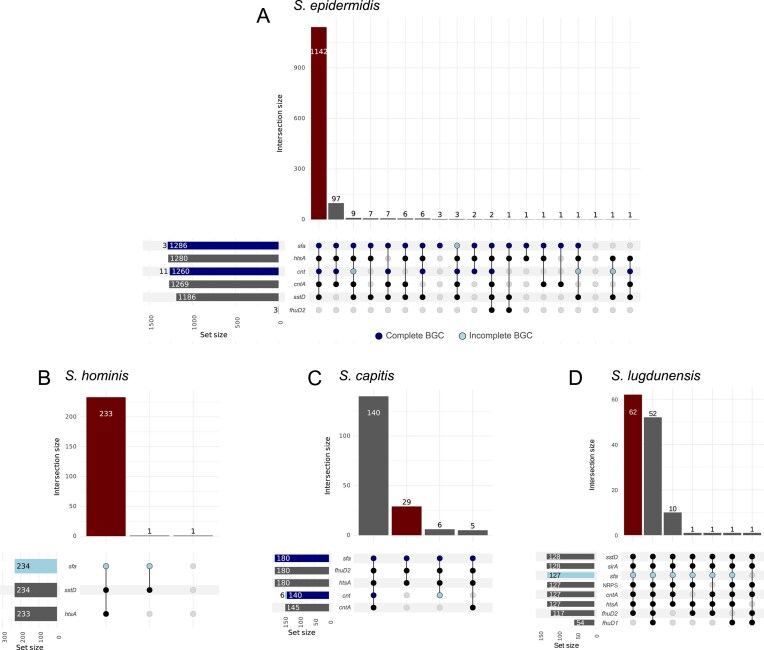
UpSet plots for the strain analysis on four selected species: (**A**) *S. epidermidis*, (**B**) *S. hominis*, (**C**) *S. capitis*, and (**D**) *S. lugdunensis*. The BGCs are classified as complete (dark blue) if they show $100\%$ similarity to the reference BGCs in the MIBiG database. Else, they are classified as incomplete (light blue). A lipoprotein homolog is detected for a strain if the similarity to the used reference is higher than $50\%$.

For *S. epidermidis*, the majority of strains (1142 of 1292 analyzed genomes) mirrored the profile of the GTDB reference strain, encoding both production and uptake systems for SF-A and STP and the presence of *sstD* (Fig. 6A). However, certain groups of isolates showed different profiles. Ninety-seven isolates lacked *sstD*, suggesting the inability of these strains to use catecholate-type siderophores. Additionally, individual isolates showed single or multiple genes being compromised, suggesting strain-level differences in their abilities to produce or consume metallophores.

Additionally, we looked for evidence for the development of divergent receptor alleles within *S. epidermidis*. Comparison of the full-length HstA protein sequences showed a high degree of conservation (69.6% to 99.2% identity to the reference allele of *S. aureus*) (Supplementary Fig. S4a). However, when considering only the residues known to be involved in SF-A binding, the conservation increased to 100% in 99.9% of isolates (Supplementary Fig. S4b). The strains carrying alterations showed truncated alleles, suggesting inactivated proteins or inaccurate assemblies. Altogether, this analysis suggests that the receptors bind SF-A in a conserved manner, and direct evidence that the receptors develop to accommodate putative SF-A variants is not apparent.

The *S. hominis* strains showed high conservation regarding their ability to produce or secrete metallophores with only 2 out of 235 sequenced isolates diverging from the GTDB reference strain by lacking individual systems (Fig. 6B).

In *S. capitis*, only 29 of the 180 strains showed the gene combination of the GTDB reference strain, while the majority of strains (140) appear proficient in STP production and acquisition. This suggests that the representative strain defined by the GTDB (GCF_002902325.1) is poorly representing the species in this regard (Fig. 6C).

Two almost equal groups of strains were identified in *S. lugdunensis*. While 62 of 128 analyzed strains showed identical traits as the reference strain, 52 strains encoded an additional FhuD1 homologue, suggesting an increased ability to acquire hydroxamate-type siderophores (Fig. 6D). In addition, 10 strains did not encode any *FhuD* protein, and three strains showed individual combinations of gene inactivations. More interestingly, the NRPS system putatively encoding a metallophore of unknown function was identified in 127 strains, showing a conserved trait across multiple *S. lugdunensis* strains.

## Conclusion

This study provides a systematic overview of metallophore synthesis and uptake potential across the *Staphylococcus* genus, revealing the diversity at both species and strain levels, based on similar approaches applied to other species [48–50]. Our analysis shows that SF-A biosynthesis is a widespread trait among staphylococci. However, multiple species harbor truncations within the *sfa* locus, likely reflecting adaptations that favor metallophore scavenging rather than the metabolic cost of siderophore production. Furthermore, it also suggests the functional replacement of SF-A, as seen in the phylogenetic clade with a novel but conserved NRPS-independent metallophore-related BGC of yet unknown function. Future analyses of this yet uncharacterized BGC could focus on its experimental validation, as well as on the application of novel bioinformatic methods that focus on the characterization of the chemical structure of the produced metabolite [51].

In contrast, SF-B and STP production is restricted to a subset of species, with examples of gene loss or inactivation (e.g. in *S. durrellii*) driving both species- and strain-level variation. Our data also confirm some occurrences of metallophore cheating, where species retained uptake systems without maintaining the corresponding biosynthesis genes. As a clear representative of the cheater species, *S. lugdunensis* displays an extensive repertoire of metallophore receptors despite lacking the capacity to synthesize SF-A, SF-B, or STP itself, suggesting a strong dependence on the metabolic output of other community members.

While our primary focus was on SF-A, SF-B, and STP, the presence of another siderophore, *aureochelin*, has been suggested [52]. However, the molecule is not characterized, and BGCs are not known. In our study, we also did not identify any BGC corresponding to this compound. This likely reflects current limitations in reference databases, such as the MIBiG database, which lack entries for these siderophores and thus prevent their detection by tools like antiSMASH. A similar issue applies to the NRPS system in *S. lugdunensis*, previously suggested to have iron-binding potential [31]. However, due to the absence of a known reference BGC, this system is annotated solely as an unclassified NRPS cluster, missing its putative function as a metallophore. Close inspection of the antiSMASH entry along with knowledge regarding the siderophore equibactin was needed to associate the NRPS with metallophore activity. Accordingly, our analysis might underestimate the occurrence of metallophore biosynthesis gene clusters. Similar problems associated with the bioinformatic mining of genomes for siderophore BGCs have also been reported by others [49, 51, 53].

Our study has analyzed 77 representatives of the *Staphylococci* genus, with a detailed analysis of four species, with a total analysis of over 1800 strains for their potential of metallophore synthesis and uptake. For three of the in-depth strain analyses, we identify that the representatives reflected a similar profile to most of the analyzed strains. Nevertheless, we have also emphasized how for some strains a strain dependency is observed, such as in the cases of *S. urelyticus, S. capitis*, and *S. lugdunensis*. Additional strain-level analyses of other specific species can provide additional insight into intra-species diversity. This phenomenon is most likely of immense ecological relevance. The emergence of cheaters put the biosynthesis and receptor genes under constant evolutionary pressure to create privatized siderophores [11]. This phenomenon is well studied, for example, in *Vibrionaceae* [48] or *Pseudomonadaceae* [42]. In the latter genus, the advanced understanding of siderophore modification and associated receptor variation has allowed accurate prediction of interactions on the strain level. Our study shows that cheaters specialized in the acquisition of SF-A, SF-B, and STP produced by other cells of the same or of another species seem prominent within the genus *Staphylococcus*, suggesting conserved ecological principles. Accordingly, it is tempting to speculate that also metallophore variants and associated variants of HtsA, SirA, and CntA might exist between staphylococcal species and strains. However, to the best of our knowledge, this is not described today, and further experimental evidence will be needed to assess this.

While we found divergence of the full-length receptor alleles, the amino acid residues known to be crucial for metallophore-binding were, with few exceptions, conserved between species. Additionally, the crucial amino acids of the HtsA protein showed perfect conservation between hundreds of *S. epidermidis* isolates. This suggests that diversification of SF-A and the associated receptors to create privatized variants might not be a prominent phenomenon. However, further biochemical characterization of SF-A derived from diverse species/strains is needed to support this hypothesis.

Moreover, our analyses investigated the uptake potential with respect to the similarity of the membrane-anchored receptors. However, the metallophore uptake mechanisms rely on further proteins, such as ABC transporters and ATPases [14, 16, 17]. Hence, further analyses could expand on this study to identify the diversity of a complete uptake system.

In summary, our study provides a comprehensive description of metallophore-related systems in staphylococcal species, revealing both conserved features and diverse potentials. These findings lay the foundation for future experimental work to characterize novel BGC products and to further investigate the ecological and clinical implications of metallophore diversity, metabolic interdependencies, and cheating strategies within this genus.

## Supplementary Material

lqaf183_Supplemental_Files

## Data Availability

The scripts used for the data acquisition and processing, as well as for the generation of Figs 2 and 6, can be found via Zenodo at https://doi.org/10.5281/zenodo.17201851. The accession codes of all 1928 assemblies throughout this study can be found in the repository as well as a supplementary file.
