# Correction to ‘A landscape of metallophore synthesis and uptake potential of the genus *Staphylococcus’*

**DOI:** 10.1093/nargab/lqag091

**Published:** 2026-07-30

This is a correction to: Mathias Witte Paz, Alina Bitzer, Kay Nieselt, Simon Heilbronner, A landscape of metallophore synthesis and uptake potential of the genus *Staphylococcus, NAR Genomics and Bioinformatics*, Volume 7, Issue 4, December 2025, lqaf183, https://doi.org/10.1093/nargab/lqaf183

In the originally published version of this manuscript, a statement was omitted from the ‘Funding’ section.

The following acknowledgement has been added: “We acknowledge support from the Open Access Publication Fund of the University of Tübingen”.

