# Supplementary material for: A landscape of metallophore synthesis and uptake potential of the genus Staphylococcus
Source: NAR Genom Bioinform. 2025 Dec 17;7(4):lqaf183. doi: 10.1093/nargab/lqaf183 (PMC12709192; doi:10.1093/nargab/lqaf183)
Supplement: lqaf183_Supplemental_Files [file lqaf183_supplemental_files.zip › Supplementary_revised.pdf]

Supplementary material for "A landscape of metallophore synthesis and uptake potential of the genus *Staphylococcus*"

Mathias Witte Paz, Alina Bitzer, Kay Nieselt & Simon Heilbronner

Supplementary Table S1: Overview of species and assemblies used in the analysis. The taxonomic classification from the NCBI and the GTDB is provided.

NCBI Accession Code	NCBI classification	GTDB classification
GCA_000433035.1	*Staphylococcus sp. CAG:324*	*CAG-313 sp000433035*
GCA_020544305.1	*Staphylococcus taiwanensis*	*Staphylococcus taiwanensis*
GCA_023665865.1	*Staphylococcus sp.*	*Duncaniella sp023665865*
GCA_945870555.1	*Staphylococcus epidermidis*	UBA970 sp945870555
GCF_000010125.1	*Staphylococcus saprophyticus* subsp. saprophyticus ATCC 15305 = NCTC 7292	*Staphylococcus saprophyticus*
GCF_000236925.1	*Staphylococcus argenteus*	*Staphylococcus argenteus*
GCF_000298075.1	*Staphylococcus massiliensis* CCUG 559 27	*Staphylococcus massiliensis*
GCF_000816085.1	*Staphylococcus hyicus*	*Staphylococcus hyicus*
GCF_000875895.1	*Staphylococcus gallinarum*	*Staphylococcus gallinarum*
GCF_000934465.1	*Staphylococcus microti*	*Staphylococcus microti*
GCF_001027105.1	*Staphylococcus aureus*	*Staphylococcus aureus*
GCF_001224225.1	*Staphylococcus borealis*	*Staphylococcus borealis*
GCF_001431205.1	*Staphylococcus sp. NAM3COL9*	*Staphylococcus sp001431205*
GCF_001500315.1	*Staphylococcus auricularis*	*Staphylococcus auricularis*
GCF_001618885.1	*Staphylococcus condimenti*	*Staphylococcus condimenti*
GCF_001792775.2	*Staphylococcus pseudintermedi us*	*Staphylococcus pseudintermedius*
GCF_002101335.1	*Staphylococcus lutrae*	*Staphylococcus lutrae*
GCF_002614725.1	*Staphylococcus edaphicus*	*Staphylococcus edaphicus*
GCF_002732165.1	*Staphylococcus xylosus*	*Staphylococcus xylosus*
GCF_002901705.1	*Staphylococcus lugdunensis*	*Staphylococcus lugdunensis*
GCF_002901845.1	*Staphylococcus hominis subsp. hominis*	*Staphylococcus hominis*
GCF_002901865.1	*Staphylococcus agnetis*	*Staphylococcus agnetis*
GCF_002901995.1	*Staphylococcus coagulans*	*Staphylococcus coagulans*
GCF_002902085.1	*Staphylococcus simiae*	*Staphylococcus simiae*
GCF_002902145.1	*Staphylococcus rostri*	*Staphylococcus rostri*
GCF_002902235.1	*Staphylococcus ureilyticus*	*Staphylococcus ureilyticus*
GCF_002902285.1	*Staphylococcus simulans*	*Staphylococcus simulans*
GCF_002902305.1	*Staphylococcus argensis*	*Staphylococcus argensis*
GCF_002902325.1	*Staphylococcus capitis subsp. capitis*	*Staphylococcus capitis*
GCF_002902345.1	*Staphylococcus arlettae*	*Staphylococcus arlettae*
GCF_002902365.1	*Staphylococcus cohnii subsp. cohnii*	*Staphylococcus cohnii*

GCF_002902385.1	*Staphylococcus intermedius NC TC 11048*	*Staphylococcus intermedius*
GCF_002902405.1	*Staphylococcus schweitzeri*	*Staphylococcus schweitzeri*
GCF_002902565.1	*Staphylococcus petrasii*	*Staphylococcus petrasii*
GCF_002902575.1	*Staphylococcus croceilyticus*	*Staphylococcus croceilyticus*
GCF_002902625.1	*Staphylococcus devriesei*	*Staphylococcus devriesei*
GCF_002902685.1	*Staphylococcus pettenkoferi*	*Staphylococcus pettenkoferi*
GCF_002902725.1	*Staphylococcus caprae*	*Staphylococcus caprae*
GCF_003012915.1	*Staphylococcus felis*	*Staphylococcus felis*
GCF_003019255.1	*Staphylococcus kloosii*	*Staphylococcus kloosii*
GCF_003019275.1	*Staphylococcus muscae*	*Staphylococcus muscae*
GCF_003697915.1	*Staphylococcus pseudoxylosus*	*Staphylococcus pseudoxylosus*
GCF_003718735.1	*Staphylococcus debuckii*	*Staphylococcus debuckii*
GCF_003970495.1	*Staphylococcus pasteuri*	*Staphylococcus pasteuri*
GCF_004785665.1	*Staphylococcus pragensis*	*Staphylococcus pragensis*
GCF_006094395.1	*Staphylococcus haemolyticus*	*Staphylococcus haemolyticus*
GCF_006742205.1	*Staphylococcus epidermidis*	*Staphylococcus epidermidis*
GCF_010365305.1	*Staphylococcus sp. MI 10-1553*	*Staphylococcus sp010365305*
GCF_013391405.1	*Staphylococcus sp. GSSP0090*	*Staphylococcus sp013391405*
GCF_013463155.1	*Staphylococcus sp. 17KM0847*	*Staphylococcus sp013463155*
GCF_014635045.1	*Staphylococcus nepalensis*	*Staphylococcus nepalensis*
GCF_015594545.1	*Staphylococcus durrellii*	*Staphylococcus durrellii*
GCF_015775975.1	*Staphylococcus lloydii*	*Staphylococcus lloydii*
GCF_016238445.1	*Staphylococcus canis*	*Staphylococcus canis*
GCF_016238465.1	*Staphylococcus caledonicus*	*Staphylococcus caledonicus*
GCF_017583065.1	*Staphylococcus shinii*	*Staphylococcus shinii*
GCF_020883535.1	*Staphylococcus ratti*	*Staphylococcus ratti*
GCF_022346615.1	*Staphylococcus sp. ACRSN*	*Staphylococcus sp022346615*
GCF_022493055.1	*Staphylococcus roterodami*	*Staphylococcus singaporensis*
GCF_022815905.2	*Staphylococcus sp. NRL 16/872*	*Staphylococcus sp022815905*
GCF_024814435.1	*Staphylococcus americanisciur i*	*Staphylococcus americanisciuri*
GCF_025519785.1	*Staphylococcus marylandisciur i*	*Staphylococcus marylandisciuri*
GCF_025558425.1	*Staphylococcus sp. IVB6240*	*Staphylococcus sp025558425*
GCF_025558585.1	*Staphylococcus sp. IVB6214*	*Staphylococcus sp025558585*
GCF_026659735.1	*Staphylococcus pettenkoferi*	*Staphylococcus sp943908435*
GCF_029024625.1	*Staphylococcus chromogenes*	*Staphylococcus chromogenes*
GCF_029024945.1	*Staphylococcus succinus*	*Staphylococcus succinus*
GCF_029024965.1	*Staphylococcus equorum*	*Staphylococcus equorum*
GCF_900097965.1	*Staphylococcus caeli*	*Staphylococcus caeli*
GCF_900183575.1	*Staphylococcus cornubiensis*	*Staphylococcus cornubiensis*
GCF_900186985.1	*Staphylococcus piscifermentan s*	*Staphylococcus piscifermentans*
GCF_900458435.1	*Staphylococcus carnosus*	*Staphylococcus carnosus*
GCF_900458815.1	*Staphylococcus saccharolyticu s*	*Staphylococcus saccharolyticus*
GCF_900458895.1	*Staphylococcus schleiferi*	*Staphylococcus schleiferi*
GCF_900636325.1	*Staphylococcus delphini*	*Staphylococcus delphini*
GCF_900636385.1	*Staphylococcus warneri*	*Staphylococcus warneri*
GCF_943737015.1	*Staphylococcus sp. Marseille- Q5304*	*Staphylococcus sp943737015*

Supplementary Table S2: Overview of additional strains used to assess the variability of the `antiSMASH` results within selected species.

**Analysed for metabolite**	**Species**	**Accession Code**	**Similarity to annotated BGC in %**			**Number of uncharacterized BGC**
			**SF-A**	**SF-B**	**STP**	
SF-A	***S. argensis***	GCF_007671915.1	75	0	0	1
		GCF_007682325.1	75	0	0	1
		GCF_007682285.1	75	0	0	1
		GCF_002902305.1	75	0	0	1
		GCF_026740095.1	75	0	0	1
	S. equorum	GCF_029531845.1	75	100	0	0
		GCF_030249045.1	75	100	0	0
		GCF_033447145.1	50	100	0	0
		GCF_001747785.1	75	100	0	0
		GCF_001747895.1	75	100	0	0
	S. pettenkoferi	GCF_002884615.1	75	0	0	1
		GCF_026660515.1	75	0	0	1
		GCF_007678805.1	75	0	0	1
		GCF_026659915.1	75	0	0	1
		GCF_030218345.1	75	0	0	1

Supplementary Table S3: Analysis of binding site conservation of *htsA*, *sirA* and *cntA*.

Position	Amino Acid	Identical	Total Sequences	Identical %
		htsA		
86	R	46	67	68.66
104	R	67	67	100.00
126	R	67	67	100.00
203	K	46	67	68.66
209	H	67	67	100.00
239	Y	67	67	100.00
299	R	67	67	100.00
304	R	46	67	68.66
306	R	67	67	100.00
		sirA		
81	W	19	19	100.00
125	R	19	19	100.00
144	T	18	19	94.74
201	R	19	19	100.00
206	R	18	19	94.74
208	Y	19	19	100.00
304	N	19	19	100.00
		cntA		
52	Y	29	29	100.00
128	W	29	29	100.00
165	R	29	29	100.00
250	R	29	29	100.00
418	R	29	29	100.00
431	W	29	29	100.00
435	Y	29	29	100.00
448	N	13	29	44.83
522	Y	29	29	100.00

Sequence	86 104 126 203 209 239 299 304 306
**AA076_RS11070|GCF_001027105.1|Staphylococcus_aureus**	RRRKHYRRR
SAMSHR1132_RS10715|GCF_000236925.1|Staphylococcus_argenteus	....
CD116_RS04275|GCF_002902405.1|Staphylococcus_schweitzeri	....
ML436_RS11065|GCF_022493055.1|Staphylococcus_singaporensis	....
CD113_RS04085|GCF_002902085.1|Staphylococcus_simiae	....
DYE57_RS03465|GCF_900458815.1|Staphylococcus_saccharolyticus	....
FNL83_RS03625|GCF_006742205.1|Staphylococcus_epidermidis	....
CD041_RS06935|GCF_002901705.1|Staphylococcus_lugdunensis	....
CD038_RS05115|GCF_002902325.1|Staphylococcus_capitis	....
CD155_RS01715|GCF_002902725.1|Staphylococcus_caprae	....
EL082_RS03405|GCF_900636385.1|Staphylococcus_warneri	....
CD121_RS07970|GCF_003970495.1|Staphylococcus_pasteuri	....
HS848_RS09670|GCF_016238465.1|Staphylococcus_caledonicus	....
CD147_RS08050|GCF_002902625.1|Staphylococcus_devriesei	....
CD128_RS05235|GCF_002902575.1|Staphylococcus_croceilyticus	....
EQ029_RS03740|GCF_006094395.1|Staphylococcus_haemolyticus	....
CD127_RS03940|GCF_002902565.1|Staphylococcus_petrasii	....
MT340_RS03715|GCF_022815905.2|Staphylococcus_sp022815905	....
E2558_RS07710|GCF_004785665.1|Staphylococcus_pragensis	....
AK212_RS00065|GCF_001224225.1|Staphylococcus_borealis	....
CD034_RS09125|GCF_002901845.1|Staphylococcus_hominis	....
CD112_RS00655|GCF_002902285.1|Staphylococcus_simulans	....
ISP08_RS03715|GCF_015775975.1|Staphylococcus_lloydii	....
NGI30_RS02145|GCF_943737015.1|Staphylococcus_sp943737015	....
BTJ66_RS03620|GCF_002614725.1|Staphylococcus_edaphicus	....
SH09_RS07565|GCF_000875895.1|Staphylococcus_gallinarum	....
ISP02_RS03280|GCF_015594545.1|Staphylococcus_durrellii	....
C7J89_RS05050|GCF_003019255.1|Staphylococcus_kloosii	....
BJS11_RS04860|GCF_900097965.1|Staphylococcus_caeli	....
MHZ36_RS03430|GCF_022346615.1|Staphylococcus_sp022346615	....
BTM19_RS09590|GCF_002732165.1|Staphylococcus_xylosus	....
SSP_RS03455|GCF_000010125.1|Staphylococcus_saprophyticus	....
HYE69_RS04710|GCF_013391405.1|Staphylococcus_sp013391405	....
D9V42_RS10400|GCF_003697915.1|Staphylococcus_pseudoxylosus	....
CKV71_RS04100|GCF_900186985.1|Staphylococcus_piscifermentans	....
A4G25_RS11725|GCF_001618885.1|Staphylococcus_condimenti	....
CNQ82_RS10365|GCF_003718735.1|Staphylococcus_debuckii	....
CD138_RS05400|GCF_002902385.1|Staphylococcus_intermedius	N..Q...K.
ACA31_RS09695|GCF_001431205.1|Staphylococcus_sp001431205	....
PYW31_RS03465|GCF_029024945.1|Staphylococcus_succinus	....
CCE82_RS04525|GCF_900183575.1|Staphylococcus_cornubiensis	N..Q...K.
NXS11_RS03460|GCF_024814435.1|Staphylococcus_americanisciuri	N..Q...K.
CD032_RS03660|GCF_002902365.1|Staphylococcus_cohnii	....
DYE31_RS03920|GCF_900458435.1|Staphylococcus_carnosus	....
J5E45_RS09575|GCF_017583065.1|Staphylococcus_shinii	....
APS80_RS07250|GCF_001500315.1|Staphylococcus_auricularis	....
IEU88_RS00065|GCF_014635045.1|Staphylococcus_nepalensis	....
CD122_RS02540|GCF_002902145.1|Staphylococcus_rostri	N..Q...K.
GZH82_RS09685|GCF_010365305.1|Staphylococcus_sp010365305	N..Q...K.
CD150_RS03745|GCF_002902235.1|Staphylococcus_ureilyticus	....
C7J88_RS05170|GCF_003019275.1|Staphylococcus_muscae	N..Q...K.
TP70_RS10485|GCF_000934465.1|Staphylococcus_microti	N..Q...K.
MUA88_RS08800|GCF_025558425.1|Staphylococcus_sp025558425	N..Q...K.
EL101_RS03425|GCF_900636325.1|Staphylococcus_delphini	N..Q...K.
MUA51_RS08560|GCF_025558585.1|Staphylococcus_sp025558585	N..Q...K.
CD172_RS09325|GCF_002901865.1|Staphylococcus_agnetis	N..Q...K.
BJK46_RS11185|GCF_001792775.2|Staphylococcus_pseudintermedius	N..Q...K.
SHYC_RS03330|GCF_000816085.1|Staphylococcus_hyicus	N..Q...K.
B5P37_RS02880|GCF_002101335.1|Staphylococcus_lutrae	N..Q...K.
PYW44_RS03795|GCF_029024965.1|Staphylococcus_equorum	....
C7J90_RS00570|GCF_003012915.1|Staphylococcus_felis	N..Q...K.
FGL66_RS07705|GCF_013463155.1|Staphylococcus_sp013463155	N..Q...K.
PYW36_RS03040|GCF_029024625.1|Staphylococcus_chromogenes	N..Q...K.
JM183_RS03315|GCF_900458895.1|Staphylococcus_schleiferi	N..Q...K.
A33S_RS0108695|GCF_000298075.1|Staphylococcus_massiliensis	N..E...K.
CD118_RS04190|GCF_002901995.1|Staphylococcus_coagulans	N..Q...K.
HHH54_RS09780|GCF_016238445.1|Staphylococcus_canis	N..Q...K.

Supplementary Figure S1: Multiple sequence alignment of identified homologs of *HtsA*. Visualized using `Jalview` [1]

	86	125	144	201	206	208	304
**AA076_RS00330|GCF_001027105.1|Staphylococcus_aureus**	W	R	T	R	R	Y	N
SAMSHR1132_RS00465|GCF_000236925.1|Staphylococcus_argenteus	.	.	.	.	.	.	.
ML436_RS00345|GCF_022493055.1|Staphylococcus_singaporensis	.	.	.	.	.	.	.
CD116_RS00940|GCF_002902405.1|Staphylococcus_schweitzeri	.	.	.	.	.	.	.
CD041_RS10505|GCF_002901705.1|Staphylococcus_lugdunensis	.	.	N	.	.	.	.
CCE82_RS05305|GCF_900183575.1|Staphylococcus_cornubiensis	.	.	.	.	.	.	.
BJK46_RS05250|GCF_001792775.2|Staphylococcus_pseudintermedius	.	.	.	.	.	.	.
EL101_RS11370|GCF_900636325.1|Staphylococcus_delphini	.	.	.	.	.	.	.
CD138_RS02245|GCF_002902385.1|Staphylococcus_intermedius	.	.	.	.	.	.	.
GZH82_RS11655|GCF_010365305.1|Staphylococcus_sp010365305	.	.	.	.	.	.	.
CD118_RS08195|GCF_002901995.1|Staphylococcus_coagulans	.	.	.	.	.	.	.
JM183_RS00365|GCF_900458895.1|Staphylococcus_schleiferi	.	.	.	.	.	.	.
CD036_RS07075|GCF_002902345.1|Staphylococcus_arlettae	.	.	.	.	.	.	.
B5P37_RS07585|GCF_002101335.1|Staphylococcus_lutrae	.	.	.	.	.	.	.
PYW44_RS00580|GCF_029024965.1|Staphylococcus_equorum	.	.	.	.	.	.	.
LN051_RS00225|GCF_020883535.1|Staphylococcus_ratti	.	.	.	.	.	.	.
CD172_RS11315|GCF_002901865.1|Staphylococcus_agnetis	.	.	.	.	.	.	.
SHYC_RS00645|GCF_000816085.1|Staphylococcus_hyicus	.	.	.	.	.	.	.
PYW36_RS00295|GCF_029024625.1|Staphylococcus_chromogenes	.	.	.	.	C	.	.

Supplementary Figure S2: Multiple sequence alignment of identified homologs of *SirA*. Visualized using `Jalview` [1]

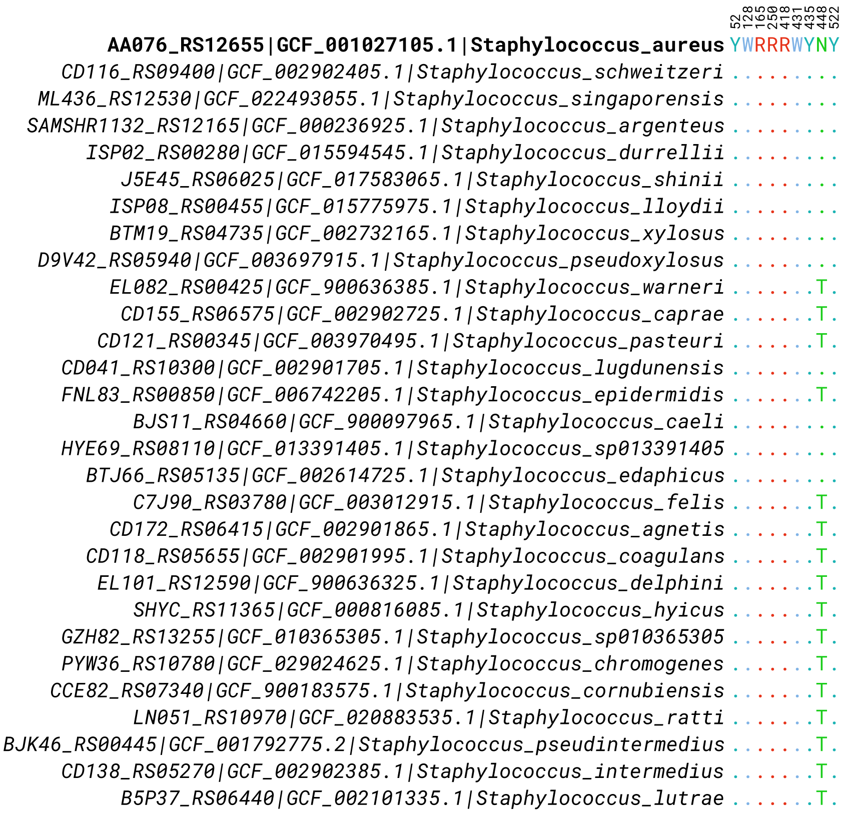

Supplementary Figure S3: Multiple sequence alignment of identified homologs of *CntA*. Visualized using `Jalview` [1]

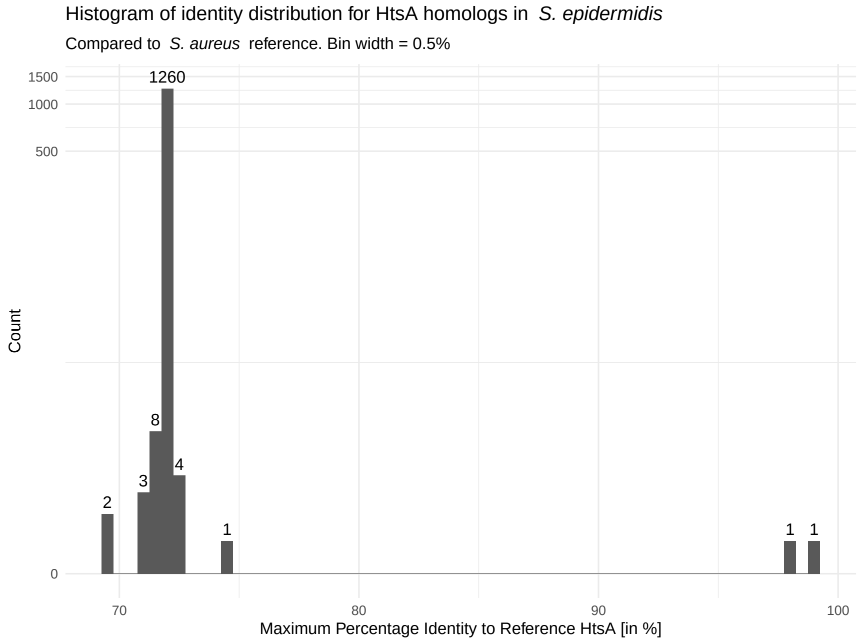

(a) Accounts full length sequence for the comparison.

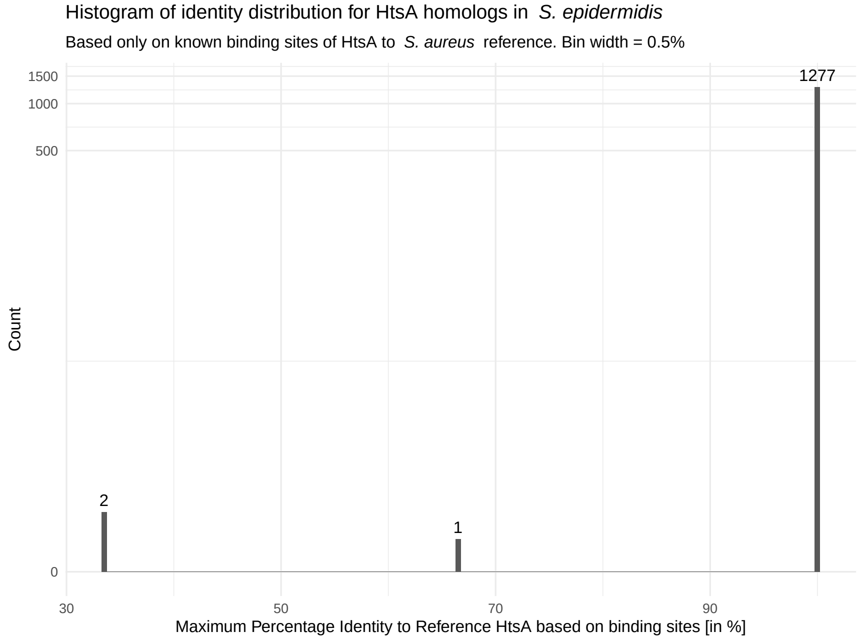

(b) Accounts only for known binding sites.

Supplementary Figure S4: Distribution of sequence identify of HtsA homologs in *S. epidermidis* when compared to the reference of *S. aureus*.

References

[1] A. M. Waterhouse, J. B. Procter, D. M. A. Martin, M. Clamp, and G. J. Barton. Jalview Version 2—a multiple sequence alignment editor and analysis workbench. *Bioinformatics*, 25(9):1189–1191, 2009.
